# Supplementary material for: Molecular and Serological Detection of Anaplasma phagocytophilum in Dogs from Germany (2008–2020)
Source: Animals (Basel). 2023 Feb 17;13(4):720. doi: 10.3390/ani13040720 (PMC9952382; doi:10.3390/ani13040720)
Supplement: Supplementary file 1 [file animals-13-00720-s001.zip › animals-2158697-supplementary.pdf]

Baden-Wuerttemberg: PCR

Year * PCR[a]

			PCR negative	PCR positive	total
Year	2008	N	81	1	82
		%	98.8%	1.2%	100.0%
	2009	N	113	7	120
		%	94.2%	5.8%	100.0%
	2010	N	120	5	125
		%	96.0%	4.0%	100.0%
	2011	N	115	6	121
		%	95.0%	5.0%	100.0%
	2012	N	150	11	161
		%	93.2%	6.8%	100.0%
	2013	N	110	3	113
		%	97.3%	2.7%	100.0%
	2014	N	139	3	142
		%	97.9%	2.1%	100.0%
	2015	N	132	5	137
		%	96.4%	3.6%	100.0%
	2016	N	195	10	205
		%	95.1%	4.9%	100.0%
	2017	N	283	6	289
		%	97.9%	2.1%	100.0%
	2018	N	309	11	320
		%	96.6%	3.4%	100.0%
	2019	N	398	13	411
		%	96.8%	3.2%	100.0%
	2020	N	624	37	661
		%	94.4%	5.6%	100.0%
Total		N	2,769	118	2,887
		%	95.9%	4.1%	100.0%

a. Federal State = Baden-Wuerttemberg

Baden-Wuerttemberg: IFAT/ELISA

Year * IFAT/ELISA[a]

			IFAT/ELISA		
			negative	positive	total
Year	2008	N	278	39	317
		%	87.7%	12.3%	100.0%
	2009	N	382	101	483
		%	79.1%	20.9%	100.0%
	2010	N	373	93	466
		%	80.0%	20.0%	100.0%
	2011	N	401	108	509
		%	78.8%	21.2%	100.0%
	2012	N	437	113	550
		%	79.5%	20.5%	100.0%
	2013	N	435	120	555
		%	78.4%	21.6%	100.0%
	2014	N	582	92	674
		%	86.4%	13.6%	100.0%
	2015	N	592	61	653
		%	90.7%	9.3%	100.0%
	2016	N	644	101	745
		%	86.4%	13.6%	100.0%
	2017	N	538	279	817
		%	65.9%	34.1%	100.0%
	2018	N	643	305	948
		%	67.8%	32.2%	100.0%
	2019	N	697	274	971
		%	71.8%	28.2%	100.0%
	2020	N	855	425	1,280
		%	66.8%	33.2%	100.0%
Gesamt		N	6,857	2,111	8,968
		%	76.5%	23.5%	100.0%

a. Federal State = Baden-Wuerttemberg

Bavaria: PCR

Year * PCR[a]

			PCR		
			negative	positive	total
Year	2008	N	75	7	82
		%	91.5%	8.5%	100.0%
	2009	N	137	8	145
		%	94.5%	5.5%	100.0%
	2010	N	109	8	117
		%	93.2%	6.8%	100.0%
	2011	N	86	5	91
		%	94.5%	5.5%	100.0%
	2012	N	138	5	143
		%	96.5%	3.5%	100.0%
	2013	N	179	5	184
		%	97.3%	2.7%	100.0%
	2014	N	221	9	230
		%	96.1%	3.9%	100.0%
	2015	N	140	11	151
		%	92.7%	7.3%	100.0%
	2016	N	172	1	173
		%	99.4%	0.6%	100.0%
	2017	N	279	14	293
		%	95.2%	4.8%	100.0%
	2018	N	305	14	319
		%	95.6%	4.4%	100.0%
	2019	N	365	21	386
		%	94.6%	5.4%	100.0%
	2020	N	376	31	407
		%	92.4%	7.6%	100.0%
Total		N	2,582	139	2,721
		%	94.9%	5.1%	100.0%

a. Federal State = Bavaria

Bavaria: IFAT/ELISA

Year * IFAT/ELISA[a]

			IFAT/ELISA		
			negative	positive	total
Year	2008	N	277	95	372
		%	74.5%	25.5%	100.0%
	2009	N	256	143	399
		%	64.2%	35.8%	100.0%
	2010	N	210	114	324
		%	64.8%	35.2%	100.0%
	2011	N	319	156	475
		%	67.2%	32.8%	100.0%
	2012	N	334	185	519
		%	64.4%	35.6%	100.0%
	2013	N	325	198	523
		%	62.1%	37.9%	100.0%
	2014	N	439	143	582
		%	75.4%	24.6%	100.0%
	2015	N	451	93	544
		%	82.9%	17.1%	100.0%
	2016	N	446	129	575
		%	77.6%	22.4%	100.0%
	2017	N	407	314	721
		%	56.4%	43.6%	100.0%
	2018	N	414	286	700
		%	59.1%	40.9%	100.0%
	2019	N	369	286	655
		%	56.3%	43.7%	100.0%
	2020	N	457	369	826
		%	55.3%	44.7%	100.0%
Total		N	4,704	2,511	7,215
		%	65.2%	34.8%	100.0%

a. Federal State = Bavaria

Berlin-Brandenburg: PCR

Year * PCR[a]

			PCR		
			negative	positive	total
Year	2008	N	24	2	26
		%	92.3%	7.7%	100.0%
	2009	N	51	1	52
		%	98.1%	1.9%	100.0%
	2010	N	59	2	61
		%	96.7%	3.3%	100.0%
	2011	N	92	10	102
		%	90.2%	9.8%	100.0%
	2012	N	133	4	137
		%	97.1%	2.9%	100.0%
	2013	N	186	9	195
		%	95.4%	4.6%	100.0%
	2014	N	247	14	261
		%	94.6%	5.4%	100.0%
	2015	N	247	24	271
		%	91.1%	8.9%	100.0%
	2016	N	305	32	337
		%	90.5%	9.5%	100.0%
	2017	N	321	42	363
		%	88.4%	11.6%	100.0%
	2018	N	339	33	372
		%	91.1%	8.9%	100.0%
	2019	N	389	34	423
		%	92.0%	8.0%	100.0%
	2020	N	414	24	438
		%	94.5%	5.5%	100.0%
Total		N	2,807	231	3,038
		%	92.4%	7.6%	100.0%

a. Federal State = Berlin-Brandenburg

Berlin-Brandenburg: IFAT/ELISA

Year * IFAT/ELISA[a]

			IFAT/ELISA		
			negative	positive	total
Year	2008	N	27	1	28
		%	96.4%	3.6%	100.0%
	2009	N	43	8	51
		%	84.3%	15.7%	100.0%
	2010	N	53	30	83
		%	63.9%	36.1%	100.0%
	2011	N	78	45	123
		%	63.4%	36.6%	100.0%
	2012	N	133	51	184
		%	72.3%	27.7%	100.0%
	2013	N	145	64	209
		%	69.4%	30.6%	100.0%
	2014	N	240	47	287
		%	83.6%	16.4%	100.0%
	2015	N	242	46	288
		%	84.0%	16.0%	100.0%
	2016	N	256	66	322
		%	79.5%	20.5%	100.0%
	2017	N	230	151	381
		%	60.4%	39.6%	100.0%
	2018	N	251	148	399
		%	62.9%	37.1%	100.0%
	2019	N	323	186	509
		%	63.5%	36.5%	100.0%
	2020	N	392	219	611
		%	64.2%	35.8%	100.0%
Total		N	2,413	1,062	3,475
		%	69.4%	30.6%	100.0%

a. Federal State = Berlin-Brandenburg

Hesse: PCR

Year * PCR[a]

			PCR		
			negative	positive	total
Year	2008	N	82	4	86
		%	95.3%	4.7%	100.0%
	2009	N	128	6	134
		%	95.5%	4.5%	100.0%
	2010	N	133	1	134
		%	99.3%	0.7%	100.0%
	2011	N	102	3	105
		%	97.1%	2.9%	100.0%
	2012	N	107	7	114
		%	93.9%	6.1%	100.0%
	2013	N	95	2	97
		%	97.9%	2.1%	100.0%
	2014	N	130	5	135
		%	96.3%	3.7%	100.0%
	2015	N	140	7	147
		%	95.2%	4.8%	100.0%
	2016	N	224	7	231
		%	97.0%	3.0%	100.0%
	2017	N	252	9	261
		%	96.6%	3.4%	100.0%
	2018	N	295	12	307
		%	96.1%	3.9%	100.0%
	2019	N	301	6	307
		%	98.0%	2.0%	100.0%
	2020	N	433	5	438
		%	98.9%	1.1%	100.0%
Total		N	2,422	74	2,496
		%	97.0%	3.0%	100.0%

a. Federal State = Hesse

Hesse: IFAT/ELISA

Year * IFAT/ELISA[a]

			IFAT/ELISA		
			negative	positive	total
Year	2008	N	325	53	378
		%	86.0%	14.0%	100.0%
	2009	N	381	112	493
		%	77.3%	22.7%	100.0%
	2010	N	286	98	384
		%	74.5%	25.5%	100.0%
	2011	N	387	143	530
		%	73.0%	27.0%	100.0%
	2012	N	477	143	620
		%	76.9%	23.1%	100.0%
	2013	N	507	113	620
		%	81.8%	18.2%	100.0%
	2014	N	759	107	866
		%	87.6%	12.4%	100.0%
	2015	N	866	70	936
		%	92.5%	7.5%	100.0%
	2016	N	882	98	980
		%	90.0%	10.0%	100.0%
	2017	N	765	319	1,084
		%	70.6%	29.4%	100.0%
	2018	N	616	211	827
		%	74.5%	25.5%	100.0%
	2019	N	761	215	976
		%	78.0%	22.0%	100.0%
	2020	N	784	261	1,045
		%	75.0%	25.0%	100.0%
Total		N	7,796	1,943	9,739
		%	80.0%	20.0%	100.0%

a. Federal State = Hesse

Lower Saxony/Bremen: PCR

Year * PCR[a]

			PCR		
			negative	positive	total
Year	2008	N	96	6	102
		%	94.1%	5.9%	100.0%
	2009	N	147	6	153
		%	96.1%	3.9%	100.0%
	2010	N	128	4	132
		%	97.0%	3.0%	100.0%
	2011	N	148	6	154
		%	96.1%	3.9%	100.0%
	2012	N	172	4	176
		%	97.7%	2.3%	100.0%
	2013	N	188	8	196
		%	95.9%	4.1%	100.0%
	2014	N	243	20	263
		%	92.4%	7.6%	100.0%
	2015	N	318	21	339
		%	93.8%	6.2%	100.0%
	2016	N	405	24	429
		%	94.4%	5.6%	100.0%
	2017	N	466	33	499
		%	93.4%	6.6%	100.0%
	2018	N	588	34	622
		%	94.5%	5.5%	100.0%
	2019	N	563	38	601
		%	93.7%	6.3%	100.0%
	2020	N	632	43	675
		%	93.6%	6.4%	100.0%
Total		N	4,094	247	4,341
		%	94.3%	5.7%	100.0%

a. Federal State = Lower Saxony/Bremen

Lower Saxony/Bremen: IFAT/ELISA

Year * IFAT/ELISA[a]

			IFAT/ELISA		
			negative	positive	total
Year	2008	N	338	85	423
		%	79.9%	20.1%	100.0%
	2009	N	403	175	578
		%	69.7%	30.3%	100.0%
	2010	N	377	217	594
		%	63.5%	36.5%	100.0%
	2011	N	587	332	919
		%	63.9%	36.1%	100.0%
	2012	N	773	435	1,208
		%	64.0%	36.0%	100.0%
	2013	N	893	458	1,351
		%	66.1%	33.9%	100.0%
	2014	N	1,050	389	1,439
		%	73.0%	27.0%	100.0%
	2015	N	1,204	284	1,488
		%	80.9%	19.1%	100.0%
	2016	N	1,183	266	1,449
		%	81.6%	18.4%	100.0%
	2017	N	980	759	1,739
		%	56.4%	43.6%	100.0%
	2018	N	1,078	812	1,890
		%	57.0%	43.0%	100.0%
	2019	N	1,235	692	1,927
		%	64.1%	35.9%	100.0%
	2020	N	1,222	829	2,051
		%	59.6%	40.4%	100.0%
Total		N	11,323	5,733	17,056
		%	66.4%	33.6%	100.0%

a. Federal State = Lower Saxony/Bremen

Mecklenburg Western Pomerania: PCR

Year * PCR[a]

			PCR		
			negative	positive	total
Year	2008	N	8	1	9
		%	88.9%	11.1%	100.0%
	2009	N	12	0	12
		%	100.0%	0.0%	100.0%
	2010	N	6	1	7
		%	85.7%	14.3%	100.0%
	2011	N	5	1	6
		%	83.3%	16.7%	100.0%
	2012	N	13	0	13
		%	100.0%	0.0%	100.0%
	2013	N	16	0	16
		%	100.0%	0.0%	100.0%
	2014	N	23	0	23
		%	100.0%	0.0%	100.0%
	2015	N	26	1	27
		%	96.3%	3.7%	100.0%
	2016	N	50	4	54
		%	92.6%	7.4%	100.0%
	2017	N	41	1	42
		%	97.6%	2.4%	100.0%
	2018	N	54	5	59
		%	91.5%	8.5%	100.0%
	2019	N	67	4	71
		%	94.4%	5.6%	100.0%
	2020	N	70	10	80
		%	87.5%	12.5%	100.0%
Total		N	391	28	419
		%	93.3%	6.7%	100.0%

a. Federal State = Mecklenburg Western Pomerania

Mecklenburg Western Pomerania: IFAT/ELISA

Year * IFAT/ELISA[a]

			IFAT/ELISA		
			negative	positive	total
Year	2008	N	18	4	22
		%	81.8%	18.2%	100.0%
	2009	N	20	4	24
		%	83.3%	16.7%	100.0%
	2010	N	17	4	21
		%	81.0%	19.0%	100.0%
	2011	N	33	12	45
		%	73.3%	26.7%	100.0%
	2012	N	34	10	44
		%	77.3%	22.7%	100.0%
	2013	N	26	8	34
		%	76.5%	23.5%	100.0%
	2014	N	36	5	41
		%	87.8%	12.2%	100.0%
	2015	N	47	4	51
		%	92.2%	7.8%	100.0%
	2016	N	50	9	59
		%	84.7%	15.3%	100.0%
	2017	N	54	13	67
		%	80.6%	19.4%	100.0%
	2018	N	46	31	77
		%	59.7%	40.3%	100.0%
	2019	N	52	26	78
		%	66.7%	33.3%	100.0%
	2020	N	85	28	113
		%	75.2%	24.8%	100.0%
Total		N	518	158	676
		%	76.6%	23.4%	100.0%

a. Federal State = Mecklenburg Western Pomerania

North Rhine Westphalia: PCR

Year * PCR[a]

			PCR		
			negative	positive	total
Year	2008	N	264	10	274
		%	96.4%	3.6%	100.0%
	2009	N	273	11	284
		%	96.1%	3.9%	100.0%
	2010	N	297	10	307
		%	96.7%	3.3%	100.0%
	2011	N	302	12	314
		%	96.2%	3.8%	100.0%
	2012	N	320	11	331
		%	96.7%	3.3%	100.0%
	2013	N	366	11	377
		%	97.1%	2.9%	100.0%
	2014	N	421	17	438
		%	96.1%	3.9%	100.0%
	2015	N	439	13	452
		%	97.1%	2.9%	100.0%
	2016	N	478	30	508
		%	94.1%	5.9%	100.0%
	2017	N	667	23	690
		%	96.7%	3.3%	100.0%
	2018	N	595	26	621
		%	95.8%	4.2%	100.0%
	2019	N	694	28	722
		%	96.1%	3.9%	100.0%
	2020	N	771	35	806
		%	95.7%	4.3%	100.0%
Total		N	5,887	237	6,124
		%	96.1%	3.9%	100.0%

a. Federal State = North Rhine Westphalia

North Rhine Westphalia: IFAT/ELISA

Year * IFAT/ELISA[a]

			IFAT/ELISA		
			negative	positive	total
Year	2008	N	669	141	810
		%	82.6%	17.4%	100.0%
	2009	N	916	312	1,228
		%	74.6%	25.4%	100.0%
	2010	N	1,007	348	1,355
		%	74.3%	25.7%	100.0%
	2011	N	1,124	413	1,537
		%	73.1%	26.9%	100.0%
	2012	N	1,360	444	1,804
		%	75.4%	24.6%	100.0%
	2013	N	1,571	439	2,010
		%	78.2%	21.8%	100.0%
	2014	N	2,032	477	2,509
		%	81.0%	19.0%	100.0%
	2015	N	2,055	280	2,335
		%	88.0%	12.0%	100.0%
	2016	N	1,826	351	2,177
		%	83.9%	16.1%	100.0%
	2017	N	1,785	978	2,763
		%	64.6%	35.4%	100.0%
	2018	N	1,634	876	2,510
		%	65.1%	34.9%	100.0%
	2019	N	1,871	819	2,690
		%	69.6%	30.4%	100.0%
	2020	N	2,023	1,035	3,058
		%	66.2%	33.8%	100.0%
Total		N	19,873	6,913	26,786
		%	74.2%	25.8%	100.0%

a. Federal State = North Rhine Westphalia

Rhineland Palatinate: PCR

Year * PCR[a]

			PCR		
			negative	positive	total
Year	2008	N	58	2	60
		%	96.7%	3.3%	100.0%
	2009	N	82	3	85
		%	96.5%	3.5%	100.0%
	2010	N	91	5	96
		%	94.8%	5.2%	100.0%
	2011	N	73	1	74
		%	98.6%	1.4%	100.0%
	2012	N	75	1	76
		%	98.7%	1.3%	100.0%
	2013	N	76	2	78
		%	97.4%	2.6%	100.0%
	2014	N	126	2	128
		%	98.4%	1.6%	100.0%
	2015	N	108	3	111
		%	97.3%	2.7%	100.0%
	2016	N	128	2	130
		%	98.5%	1.5%	100.0%
	2017	N	170	11	181
		%	93.9%	6.1%	100.0%
	2018	N	221	4	225
		%	98.2%	1.8%	100.0%
	2019	N	235	12	247
		%	95.1%	4.9%	100.0%
	2020	N	236	7	243
		%	97.1%	2.9%	100.0%
Total		N	1,679	55	1,734
		%	96.8%	3.2%	100.0%

a. Federal State = Rhineland Palatinate

Rhineland Palatinate: IFAT/ELISA

Year * IFAT/ELISA[a]

			IFAT/ELISA		
			negative	positive	total
Year	2008	N	140	15	155
		%	90.3%	9.7%	100.0%
	2009	N	199	51	250
		%	79.6%	20.4%	100.0%
	2010	N	229	66	295
		%	77.6%	22.4%	100.0%
	2011	N	234	78	312
		%	75.0%	25.0%	100.0%
	2012	N	393	79	472
		%	83.3%	16.7%	100.0%
	2013	N	374	84	458
		%	81.7%	18.3%	100.0%
	2014	N	438	78	516
		%	84.9%	15.1%	100.0%
	2015	N	497	42	539
		%	92.2%	7.8%	100.0%
	2016	N	428	51	479
		%	89.4%	10.6%	100.0%
	2017	N	468	223	691
		%	67.7%	32.3%	100.0%
	2018	N	490	180	670
		%	73.1%	26.9%	100.0%
	2019	N	504	188	692
		%	72.8%	27.2%	100.0%
	2020	N	534	223	757
		%	70.5%	29.5%	100.0%
Total		N	4,928	1,358	6,286
		%	78.4%	21.6%	100.0%

a. Federal State = Rhineland Palatinate

Saarland: PCR

Year * PCR[a]

			PCR		
			negative	positive	total
Year	2008	N	38	3	41
		%	92,7%	7,3%	100,0%
	2009	N	41	0	41
		%	100,0%	0,0%	100,0%
	2010	N	45	2	47
		%	95,7%	4,3%	100,0%
	2011	N	53	4	57
		%	93,0%	7,0%	100,0%
	2012	N	73	4	77
		%	94,8%	5,2%	100,0%
	2013	N	68	3	71
		%	95,8%	4,2%	100,0%
	2014	N	128	5	133
		%	96,2%	3,8%	100,0%
	2015	N	52	0	52
		%	100,0%	0,0%	100,0%
	2016	N	30	1	31
		%	96,8%	3,2%	100,0%
	2017	N	32	0	32
		%	100,0%	0,0%	100,0%
	2018	N	50	0	50
		%	100,0%	0,0%	100,0%
	2019	N	68	3	71
		%	95,8%	4,2%	100,0%
	2020	N	42	3	45
		%	93,3%	6,7%	100,0%
Total			720	28	748
			96.3%	3,7%	100,0%

a. Federal State = Saarland

Saarland: IFAT/ELISA

Year * IFAT/ELISA[a]

			Antikörpernachweis		
			negative	positive	total
Year	2008	N	128	36	164
		%	78.0%	22.0%	100.0%
	2009	N	144	59	203
		%	70.9%	29.1%	100.0%
	2010	N	104	87	191
		%	54.5%	45.5%	100.0%
	2011	N	136	63	199
		%	68.3%	31.7%	100.0%
	2012	N	153	75	228
		%	67.1%	32.9%	100.0%
	2013	N	120	42	162
		%	74.1%	25.9%	100.0%
	2014	N	158	43	201
		%	78.6%	21.4%	100.0%
	2015	N	104	18	122
		%	85.2%	14.8%	100.0%
	2016	N	102	21	123
		%	82.9%	17.1%	100.0%
	2017	N	93	40	133
		%	69.9%	30.1%	100.0%
	2018	N	97	68	165
		%	58.8%	41.2%	100.0%
	2019	N	120	61	181
		%	66.3%	33.7%	100.0%
	2020	N	85	59	144
		%	59.0%	41.0%	100.0%
Total			1,544	672	2,216
			69.7%	30.3%	100.0%

a. Federal State = Saarland

Saxony: PCR

Year * PCR[a]

			PCR		
			negative	positive	total
Year	2008	N	27	3	30
		%	90.0%	10.0%	100.0%
	2009	N	86	3	89
		%	96.6%	3.4%	100.0%
	2010	N	100	2	102
		%	98.0%	2.0%	100.0%
	2011	N	55	1	56
		%	98.2%	1.8%	100.0%
	2012	N	39	6	45
		%	86.7%	13.3%	100.0%
	2013	N	49	1	50
		%	98.0%	2.0%	100.0%
	2014	N	35	2	37
		%	94.6%	5.4%	100.0%
	2015	N	53	3	56
		%	94.6%	5.4%	100.0%
	2016	N	88	6	94
		%	93.6%	6.4%	100.0%
	2017	N	93	13	106
		%	87.7%	12.3%	100.0%
	2018	N	74	9	83
		%	89.2%	10.8%	100.0%
	2019	N	121	17	138
		%	87.7%	12.3%	100.0%
	2020	N	110	10	120
		%	91.7%	8.3%	100.0%
Total		N	930	76	1,006
		%	92.4%	7.6%	100.0%

a. Federal State = Saxony

Saxony: IFAT/ELISA

Year * IFAT/ELISA[a]

			IFAT/ELISA negative	IFAT/ELISA positive	total
Year	2008	N	110	23	133
		%	82.7%	17.3%	100.0%
	2009	N	102	27	129
		%	79.1%	20.9%	100.0%
	2010	N	82	16	98
		%	83.7%	16.3%	100.0%
	2011	N	84	37	121
		%	69.4%	30.6%	100.0%
	2012	N	112	19	131
		%	85.5%	14.5%	100.0%
	2013	N	100	34	134
		%	74.6%	25.4%	100.0%
	2014	N	126	31	157
		%	80.3%	19.7%	100.0%
	2015	N	129	20	149
		%	86.6%	13.4%	100.0%
	2016	N	99	31	130
		%	76.2%	23.8%	100.0%
	2017	N	96	69	165
		%	58.2%	41.8%	100.0%
	2018	N	84	55	139
		%	60.4%	39.6%	100.0%
	2019	N	113	56	169
		%	66.9%	33.1%	100.0%
	2020	N	109	53	162
		%	67.3%	32.7%	100.0%
Total		N	1,346	471	1,817
		%	74.1%	25.9%	100.0%

a. Federal State = Saxony

Saxony-Anhalt: PCR

Year * PCR[a]

			PCR		
			negative	positive	total
Year	2008	N	12	1	13
		%	92.3%	7.7%	100.0%
	2009	N	13	0	13
		%	100.0%	0.0%	100.0%
	2010	N	24	2	26
		%	92.3%	7.7%	100.0%
	2011	N	17	1	18
		%	94.4%	5.6%	100.0%
	2012	N	11	0	11
		%	100.0%	0.0%	100.0%
	2013	N	13	0	13
		%	100.0%	0.0%	100.0%
	2014	N	20	2	22
		%	90.9%	9.1%	100.0%
	2015	N	29	1	30
		%	96.7%	3.3%	100.0%
	2016	N	35	0	35
		%	100.0%	0.0%	100.0%
	2017	N	21	2	23
		%	91.3%	8.7%	100.0%
	2018	N	34	2	36
		%	94.4%	5.6%	100.0%
	2019	N	56	3	59
		%	94.9%	5.1%	100.0%
	2020	N	82	2	84
		%	97.6%	2.4%	100.0%
Total		N	367	16	383
		%	95.8%	4.2%	100.0%

a. Federal State = Saxony-Anhalt

Saxony-Anhalt: IFAT/ELISA

Year * IFAT/ELISA[a]

			IFAT/ELISA		
			negative	positive	total
Year	2008	N	27	1	28
		%	96.4%	3.6%	100.0%
	2009	N	9	7	16
		%	56.3%	43.8%	100.0%
	2010	N	28	8	36
		%	77.8%	22.2%	100.0%
	2011	N	24	10	34
		%	70.6%	29.4%	100.0%
	2012	N	36	9	45
		%	80.0%	20.0%	100.0%
	2013	N	41	6	47
		%	87.2%	12.8%	100.0%
	2014	N	81	22	103
		%	78.6%	21.4%	100.0%
	2015	N	67	10	77
		%	87.0%	13.0%	100.0%
	2016	N	72	16	88
		%	81.8%	18.2%	100.0%
	2017	N	59	51	110
		%	53.6%	46.4%	100.0%
	2018	N	69	34	103
		%	67.0%	33.0%	100.0%
	2019	N	60	44	104
		%	57.7%	42.3%	100.0%
	2020	N	84	55	139
		%	60.4%	39.6%	100.0%
Total		N	657	273	930
		%	70.6%	29.4%	100.0%

a. Federal State = Saxony-Anhalt

Schleswig Holstein/Hamburg: PCR

Year * PCR[a]

			PCR		
			negative	positive	total
Year	2008	N	41	0	41
		%	100.0%	0.0%	100.0%
	2009	N	44	5	49
		%	89.8%	10.2%	100.0%
	2010	N	76	0	76
		%	100.0%	0.0%	100.0%
	2011	N	58	3	61
		%	95.1%	4.9%	100.0%
	2012	N	58	5	63
		%	92.1%	7.9%	100.0%
	2013	N	54	1	55
		%	98.2%	1.8%	100.0%
	2014	N	73	3	76
		%	96.1%	3.9%	100.0%
	2015	N	67	4	71
		%	94.4%	5.6%	100.0%
	2016	N	91	5	96
		%	94.8%	5.2%	100.0%
	2017	N	100	7	107
		%	93.5%	6.5%	100.0%
	2018	N	103	4	107
		%	96.3%	3.7%	100.0%
	2019	N	101	9	110
		%	91.8%	8.2%	100.0%
	2020	N	133	6	139
		%	95.7%	4.3%	100.0%
Total		N	999	52	1,051
		%	95.1%	4.9%	100.0%

a. Federal State = Schleswig-Holstein/Hamburg

Schleswig Holstein/Hamburg: IFAT/ELISA

Year * IFAT/ELISA[a]

			IFAT/ELISA negative	IFAT/ELISA positive	total
Year	2008	N	100	35	135
		%	74.1%	25.9%	100.0%
	2009	N	150	47	197
		%	76.1%	23.9%	100.0%
	2010	N	173	92	265
		%	65.3%	34.7%	100.0%
	2011	N	207	67	274
		%	75.5%	24.5%	100.0%
	2012	N	204	88	292
		%	69.9%	30.1%	100.0%
	2013	N	236	104	340
		%	69.4%	30.6%	100.0%
	2014	N	333	91	424
		%	78.5%	21.5%	100.0%
	2015	N	300	41	341
		%	88.0%	12.0%	100.0%
	2016	N	211	44	255
		%	82.7%	17.3%	100.0%
	2017	N	219	143	362
		%	60.5%	39.5%	100.0%
	2018	N	226	131	357
		%	63.3%	36.7%	100.0%
	2019	N	214	110	324
		%	66.0%	34.0%	100.0%
	2020	N	297	183	480
		%	61.9%	38.1%	100.0%
Total		N	2,870	1,176	4,046
		%	70.9%	29.1%	100.0%

a. Federal State = Schleswig-Holstein/Hamburg

Thuringia: PCR

Year * PCR[a]

			PCR		
			negative	positive	total
Year	2008	N	4	0	4
		%	100.0%	0.0%	100.0%
	2009	N	14	3	17
		%	82.4%	17.6%	100.0%
	2010	N	15	0	15
		%	100.0%	0.0%	100.0%
	2011	N	21	2	23
		%	91.3%	8.7%	100.0%
	2012	N	8	0	8
		%	100.0%	0.0%	100.0%
	2013	N	15	1	16
		%	93.8%	6.3%	100.0%
	2014	N	17	2	19
		%	89.5%	10.5%	100.0%
	2015	N	36	7	43
		%	83.7%	16.3%	100.0%
	2016	N	53	2	55
		%	96.4%	3.6%	100.0%
	2017	N	63	6	69
		%	91.3%	8.7%	100.0%
	2018	N	36	3	39
		%	92.3%	7.7%	100.0%
	2019	N	50	2	52
		%	96.2%	3.8%	100.0%
	2020	N	57	3	60
		%	95.0%	5.0%	100.0%
Total		N	389	31	420
		%	92.6%	7.4%	100.0%

a. Federal State = Thuringia

Thuringia: IFAT/ELISA

Year * IFAT/ELISA[a]

			IFAT/ELISA		
			negative	positive	total
Year	2008	N	20	3	23
		%	87.0%	13.0%	100.0%
	2009	N	23	8	31
		%	74.2%	25.8%	100.0%
	2010	N	34	14	48
		%	70.8%	29.2%	100.0%
	2011	N	69	27	96
		%	71.9%	28.1%	100.0%
	2012	N	72	18	90
		%	80.0%	20.0%	100.0%
	2013	N	68	28	96
		%	70.8%	29.2%	100.0%
	2014	N	67	9	76
		%	88.2%	11.8%	100.0%
	2015	N	78	15	93
		%	83.9%	16.1%	100.0%
	2016	N	80	25	105
		%	76.2%	23.8%	100.0%
	2017	N	69	44	113
		%	61.1%	38.9%	100.0%
	2018	N	61	46	107
		%	57.0%	43.0%	100.0%
	2019	N	73	41	114
		%	64.0%	36.0%	100.0%
	2020	N	113	61	174
		%	64.9%	35.1%	100.0%
Total		N	827	339	1,166
		%	70.9%	29.1%	100.0%

a. Federal State = Thuringia
